# Metal Levels in Crab Sentinel Species from the Mediterranean Sea: Safety and Toxicological Risk Assessment

**DOI:** 10.3390/ani16050857

**Published:** 2026-03-09

**Authors:** Clara Naccari, Gaetano Cammilleri, Davide Alfonso Cammarata, Roberta Cicala, Antonio Procopio, Vincenzo Ferrantelli, Ernesto Palma

**Affiliations:** 1 Department of Health Sciences, University “Magna Græcia” of Catanzaro, 88100 Catanzaro, Italy; procopio@unicz.it (A.P.); palma@unicz.it (E.P.); 2 Food Department, Istituto Zooprofilattico Sperimentale della Sicilia “A. Mirri”, 90129 Palermo, Italy; gaetano.cammilleri86@gmail.com (G.C.); cammdavide@gmail.com (D.A.C.); roberta_cicala@virgilio.it (R.C.); vincenzo.ferrantelli@izssicilia.it (V.F.)

**Keywords:** heavy metals and metalloids, *Eriphia verrucosa*, *Cancer pagurus*, *Pachygrapsus marmoratus*, Metal Pollution Index, crab health status, thresholds

## Abstract

The assessment of metal contamination in aquatic species represents an important tool to monitor the risk of exposure for these species in relation to marine pollution. Crabs are the sentinel species of the aquatic environment and are very sensitive to metal exposure because these inorganic contaminants can be easily absorbed into their exoskeleton and migrate into their inner tissues. The aim of this study was to assess the content of metals and metalloids (Hg, Pb, Cd, As, Cr, Mn and Ni) in species of warty crab (*Eriphia verrucosa*), brown crab (*Cancer pagurus*), and marbled crab (*Pachygrapsus marmoratus*), mainly distributed along the Mediterranean coastlines. The investigations, carried out on several parts of crabs (carapace and the related pulp, gills, claws and bronchial muscle) showed the presence of residual levels of metals and metalloids (Mn > Pb > Hg > As > Ni > Cr > Cd), correlated to the distribution of these elements in the marine environment and to the specific biological features of crabs. However, the values of Metal Pollution Index (MPI) suggested a low level of contamination of the marine ecosystem where these species were found, and the coefficient of correlation (K), a specific parameters of toxicological risk assessment, was indicative of crab health status. However, the residual levels of Hg, Pb and As above the MRL in all analyzed crab species underlined the importance of continuous monitoring studies on the safety of these sea-foods.

## Simple Summary

The assessment of metal contamination in marine species represents an important tool to monitor the environment pollution and the consequent risk of exposure for aquatic species. Among these marine species, crabs can be considered as the valid sentinel species of the marine environment due to their ability to accumulate pollutants in their tissues. In this study, the content of metals and metalloids (Hg, Pb, Cd, As, Cr, Mn and Ni) was assessed in three crab species, mainly distributed along the Mediterranean coastlines: warty crab (*Eriphia verrucosa*), brown crab (*Cancer pagurus*) and marbled crab (*Pachygrapsus marmoratus*). The analysis conducted in the carapace and the related pulp, gills, claws and bronchial muscle of crabs provided evidence of a low exposure to metals in the marine environment where the crabs lived and did not exhibit a significant risk for the health status of these species, as determined by the specific parameters of toxicological risk assessment. However, the presence of trace metals in carapace pulp needs to be monitored to determine the safety of these sea-foods.

## 1. Introduction

The health and survival of aquatic species are directly correlated to marine environmental pollution due to the presence of several inorganic and organic contaminants that are able to accumulate in the aquatic ecosystem. Among these contaminants, metals and metalloids (Hg, Cd, Pb, Cr and As) released from natural processes (geological cycles, volcanic activity, dust deposition, erosion of the terrestrial crust, etc.) and anthropogenic activities (fossil fuel combustion, waste incineration, industrial waste, agricultural practices, maritime traffic, etc.) can easily reach the marine environment and bio-accumulate and bio-magnify through the aquatic food chain [1,2]. Due to their toxicity and environmental persistence, the presence of heavy metals is particularly significant for the potential risks for marine biota. In fact, they can cause physiological alterations in tissues and organs of marine species [3,4,5], promote the production of harmful metabolic by-products and reactive oxygen species (ROS), which are responsible for oxidative stress, damage the cellular lipid membrane, impair neurotransmission, suppress immune, cause genotoxicity, etc. [6]. However, metal accumulation in marine species and its related toxicity are correlated to the physicochemical features and presence of metals in seawaters; in addition, the adsorption of mineral elements in the body and tissues of aquatic organisms depends on species-specific biological factors and interactions along the marine trophic food chain [7,8].

Several aquatic species, such as fishes, crustaceans and mollusks, are commonly used as sentinels of marine pollution in eco-toxicological studies [9,10] and, at the same time, to monitor the quality and safety of seafood [11]. Among crustaceans, crabs are benthic species important in the balance of the marine ecosystem (by controlling marine organisms, such as small fish and mollusks) and, at the same time, sensitive to the risks of marine pollution, overfishing and climatic changes [12].

Crabs, due to their longevity and abundance in the marine environment, are valid aquatic sentinels [13] because they are species with low mobility and are able to accumulate higher metal levels than fishes and other marine mammalians living in open waters [14,15]. In particular, crabs live in the bottom sediments where metals are stored, and they play a key trophic role, feeding mainly on detritus, sediments, bacteria, algae and worms; therefore, due to their food habits, they are considered as the scavengers of the marine environment [16] and, consequently, are more sensitive to metal pollution [17]. In detail, they are characterized by specific biological features, such as the presence of carapaces, claws, gills, and the other parts of exoskeleton that are rich in chitin, a natural polymer able to absorb pollutants, particularly metals [18,19]. Therefore, heavy metals and metalloids, directly adsorbed by crabs or ingested from food, water, or sediment, can be easily accumulated from chitin in the exoskeleton and migrate into the inner tissues and organs [20,21], with possible toxicological health risks for these species [19]. In addition, crabs are rich in metallothioneins, which are metal-binding proteins, with a primary role in the homeostasis of essential metals, such as Cu and Zn, and a detoxifying action against toxic metals and metalloids (Hg, Pb, Cd and As) [22,23]. Therefore, the use of crabs as sentinel species [13,24,25] provides useful information on both the health of the aquatic animals and of the marine environment.

A marine area very interesting is represented by the Mediterranean Sea, a basin highly exposed to the circulation of pollutants, due to the high urbanization of the coastlines and maritime traffic, with a significant impact on the aquatic biota [26]. The crab species mainly distributed along the Mediterranean coastlines are represented by warty crab (*Eriphia verrucosa*), brown crab (*Cancer pagurus*) and marbled crab (*Pachygrapsus marmoratus*), which are selected in this study for their relevant ecological status and/or physiological characteristics.

*Eriphia verrucosa* is commonly known as “warty crab” due to the presence of warts on its carapace and claws (similar to hairs), and it presents an oval or heart-shaped carapace of reddish-brown color. This species is distributed in the Mediterranean Sea, including the Black Sea and the Western Atlantic Ocean [27,28,29]. In spring, it migrates to shallow waters (<1 m depth), playing an important role for marine species feeding on small crabs, gastropods, bivalves and mollusks. It is able to live in the extreme environmental conditions of the rocky intertidal zone, such as variations in salinity, temperature, and humidity. Instead, it is sensitive to pollutants and thus is considered a valid bio-indicator of the aquatic environment. It also represents an important commercial seafood species in the local markets of the Mediterranean countries [30,31].

*Cancer pagurus*, commonly known as “brown crab” due to its orange-reddish-brown color, is a robust crab having an oval carapace with a characteristic “pie crust” edge and black tips on its claws. It resides on the seafloor at a water temperature of 15 °C, but in presence of ocean warming, it migrates to northern waters; in this reason, it is present in the North Sea, North Atlantic Ocean, and Mediterranean Sea. It is a carnivorous and nocturnal predator of mollusks (avoiding predators like seals and cod) [32]. This species is considered as an edible crab, being a highly appreciated seafood by the European populations [33], and it constitutes an important source of income in Northern European countries; however, being very sensitive to metals bioaccumulation, it needs to be monitored for guaranteeing consumer safety.

*Pachygrapsus marmoratus* is characterized by small dimensions and yellow marbled spots; thus, it is called “marbled crab”. This species is very abundant because the females are highly prolific and the adults are sedentary. It inhabits the rocky coasts of the Mediterranean Sea, Black Sea and Northeastern Atlantic Ocean, including Brittany, the Canary Islands and the Azores [34,35]; it feeds on hard-shelled organisms, but it is a prey of fishes, gulls, and octopuses. As a sedentary species and being sensitive to the bioaccumulation of inorganic and organic pollutants, it is considered as a valid bio-indicator of marine environmental quality. Due to its small size, it is not a commercial species, contrary to *Eriphia verrucosa* and *Cancer pagurus* that are traded in local fisheries.

The aim of this study was to assess the content of metals and metalloids (Hg, Pb, Cd, As, Cr, Mn and Ni) in three crab species (*Eriphia verrucosa*, *Cancer pagurus* and *Pachygrapsus marmoratus*) collected along the Sicilian coastlines of the Strait of Messina (Italy). A comparative study has been carried out on metal distribution in these three crab species to evaluate their health status and safety. Finally, from the results obtained, a combined assessment of the ecological and food risks has been conducted for the marine environment and seafood consumers safety.

## 2. Materials and Methods

### 2.1. Sampling

**Figure 1:**
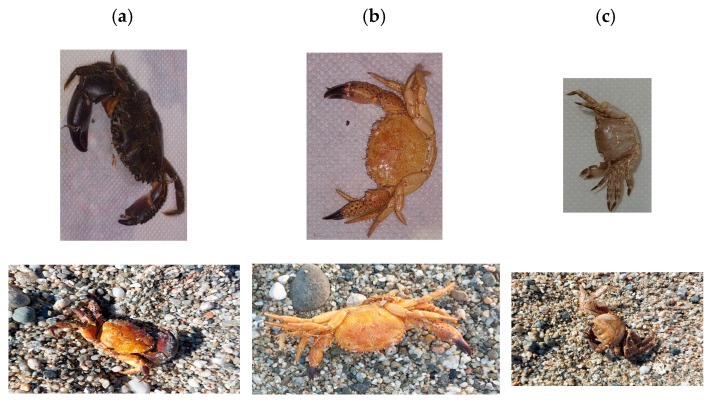
Crabs analyzed: (**a**) *Eriphia verrucosa* or warty crab, (**b**) *Cancer pagurus* or brown crab and (**c**) *Pachygrapsus marmoratus* or marbled crab.

The analysis was carried out on three crab species, identified based on their morphological features: *Eriphia verrucosa* or “warty crab”, *Cancer pagurus* or “brown crab”, and *Pachygrapsus marmoratus* or “marbled crab” (Figure 1). All crabs naturally died, were found along the Mediterranean coastlines of the Strait of Messina (Italy), and were manually collected during the autumn season. The samples had intact gills and did not show any external signs of abnormality. They were washed with distilled water to remove sand and sediments adhered to their body. Subsequently, length, weight and sex (distinguished for the inner form of carapace) were recorded (Table 1). Each crab was opened around the ventral carapace margin to collect different anatomical portions (carapace and the related pulp, claws, gills and bronchial muscle samples). Each sample was homogenized, preserved in PET containers and frozen at −20 °C until analysis.

### 2.2. Sample Preparation

The samples were subjected to acid digestion according the method of Naccari et al. (2015) [36]. Approximately 0.5 g of each sample was digested with 7 mL of HNO_3_ (65%, *v*/*v*), 1 mL of H_2_O_2_ (30%, *v*/*v*) and 1 mL of HCl (30%, *v*/*v*) in acid-prewashed PTFE vessels. All samples were digested using a closed-vessel microwave system Multiwave 3000 (Antoon Paar, Rivoly, Italy), programmed with a power of 800 W, a pressure of 80 PSI, an increased rate of 0.5 bar/s and a temperature of 180 °C. After cooling down to room temperature, the digested samples were quantitatively transferred into pre-cleaned 25 mL volumetric flasks, filtered, diluted to make up the volume with deionized water, and stored at 4 °C. Blank samples were analyzed in a similar manner. All determinations were carried out in triplicate.

### 2.3. ICP-MS Analysis

The determination of Pb, Cd, As, Cr, Mn and Ni was carried out by ICP-MS spectrometer (Thermo Scientific, Waltham, MA, USA), equipped with an auto-sampler ASX520 (Cetac Technologies Inc., Omaha, NE, USA). The ICP-MS operating conditions were set according to the method of Naccari et al. (2024) [1]. The operating conditions were: RF power, 1550 W; plasma gas flow rate, 14 L min^−1^; auxiliary gas flow rate, 0.89 L min^−1^; carrier gas flow rate 0.91 L min^−1^; helium collision gas flow rate, 4.5 mL min^−1^; spray chamber temperature, 2.70 °C; sample depth, 4.27 mm; sample introduction flow rate, 0.93 mL min^−1^; nebulizer pump, 0.1 rps; and extract lens 1 voltage, 1.5 V. Regarding data acquisition, the instrument was operated in the He KED mode to remove spectral interferences both for the low-mass and high-mass elements. Monitored isotopes were ^208^Pb, ^111^Cd, ^75^As, ^52^Cr, ^55^Mn and ^60^Ni, chosen to maximize sensitivity and to minimize interferences due to the matrix. On-line internal standards were used, i.e.,^45^Sc, ^72^Ge, ^209^Bi, and ^115^In. To integrate the peaks, 3 points for each mass and 3 replicate acquisitions were taken, using equations reported in various EPA methods and applied by instrument software (Qtegra-iCAP Q-1.5.1189.23 Windows 10^®^), containing the naturally occurring isotope ratios of elements and allowing the subtraction of isobaric or polyatomic interferences. Samples were analyzed in batches, with blank samples and known standards. Metals and metalloids quantification was carried out using the external standard method and the calibration curve constructed at five levels (range: 0.25–10 ppb for Pb, Cd, As, Cr, Mn and Ni).

### 2.4. Hg Determination

The quantification of Hg was carried out using a DMA-80 absorption spectrometer (Milestone, Middletown, CT, USA). From each crab sample, aliquots of 0.1 ± 0.001 g were weighed, put into nickel vessels, introduced into the direct analyzer and subjected to thermally decomposition at the following conditions, i.e., temperature drying: 200 °C; decomposition: 200 °C; catalysis: 650 °C; amalgam: 650 °C, with oxygen used as gas carrier (flow: 200 mL/min; pressure: 4 bar). The quality assurance protocol included calibration with ≥98.8% pure standards and the analysis of certified reference materials from proficiency tests (FCCM46-SEA7, Fapas) and duplicate samples. The calibration curves were constructed by correlating each known Hg concentration (5 concentration points from 0.050 to 2 mg/kg) with the corresponding absorbance value (λ 254 nm).

### 2.5. Method Validation

For method validation, all specific parameters were assessed (Table 2). The specificity was confirmed by the analysis of blank samples; the accuracy and precision were assessed using spiked samples and the recovery test, evaluating the mean recovery in the matrix of samples fortified at three concentration levels within the range of measurement. To check the linearity, a standard mixture at five concentration levels was analyzed for three determinations (r^2^ > 0.995). Good laboratory practice (GLP) was applied throughout the analysis.

### 2.6. Parameters to Assess Metal Pollution and Crab Health Status

The metal pollution in all three crab species was calculated with the Metal Pollution Index (MPI), a parameter which expresses the metal accumulation levels in each organ and tissue analyzed [37], using the following formula:

*MPI* = (*M*1 × *M*2 × *M*3 × *Mn*)1/*n* (1)

where M*n* represents the concentration of “*n*” metal (μg g^−1^) found in a tissue sample.

The crab health status, instead, was evaluated using the Coefficient of Condition (K), the parameter used to express the relationship between weight and length [38,39], calculated for each sample according to the following Fulton equation:

*K* = 100 × *W*/*L*^3^ (2)

where *W* is the weight (g), and *L* is the body length (cm), with a value of 1 considered as a safe level.

Considering that crabs were also recognized as seafood, the values of Estimated Daily Intake (EDI) were calculated in the edible parts, according to the following formula:

EDI = (C × IR/BW)(3)

where C is the concentration of each metal detected in crab samples (µg g^−1^), IR is the daily intake (considering an assumption of 15 g/day) [40], and BW is the body mass (70 kg for an adult).

### 2.7. Statistical Analysis

Data are expressed as mean values ± SD wet weight (w.w.) of at least three determinations. A Shapiro–Wilk test was employed to assess the normality of the data distribution. One-way analysis of variance (ANOVA) with Tukey’s post hoc pairwise comparison test was performed to test significant differences between the species examined (*p* < 0.05).

Principal component analysis (PCA) was carried out. Trace metal and metalloid data were pre-treated with Pareto-Scaling. Three different tests were conducted to assess the correct number of principal components (PCs): the Kaiser–Harris criterion, Cattell Scree test and parallel analysis. All the statistical analysis were carried out using the R 4.4.2 software using the Rcmdr plugin with the FactoMineR 2.13 package.

## 3. Results

The results confirmed the presence of all metals and metalloids analyzed in each crab sample (Table 3, Table 4 and Table 5). The highest concentrations were those of Mn in the three species (*E. verrucosa* range: 6.145–1.726 μg g^−1^; *C. pagurus* range: 5.516–0.679 μg g^−1^; *P. marmoratus* range: 2.859–1.549 μg g^−1^), followed by Pb in *E. verrucosa* (range: 1.678–0.291 μg g^−1^) and *C. pagurus* (range: 1.685–0.148 μg g^−1^) and Hg in *P. marmoratus* (range: 0.512–0.212 μg g^−1^). Instead, intermediate values were found for As (*E. verrucosa* range: 0.672–0.284 μg g^−1^; *C. pagurus* range: 0.457–0.094 μg g^−1^; *P. marmoratus* range: 0.279–0.012 μg g^−1^) and Ni (*E. verrucosa* range: 0.547–0.092 μg g^−1^; *C. pagurus* range: 0.491–0.025 μg g^−1^; *P. marmoratus* range: 0.154–0.045 μg g^−1^) while the lowest values were found for Cr (*E. verrucosa* range: 0.218–0.046 μg g^−1^; *C. pagurus* range: 0.231–0.028 μg g^−1^; *P. marmoratus* range: 0.064–0.019 μg g^−1^) and Cd (*E. verrucosa* range: 0.034–0.003 μg g^−1^; *C. pagurus* range: 0.052–0.006 μg g^−1^; *P. marmoratus* range: 0.012–0.002 μg g^−1^) in all crab samples.

In terms of metal accumulation in the three species, *E. verrucosa* and *C. pagurus* showed a similar trend with higher levels of metals analyzed in all tissues and organs than those found in *P. marmoratus*.

Considering, instead, the metal distribution and adsorption in different parts analyzed, in all the crabs species, the highest values were found in the carapace, followed by the gills and claws, whereas the lowest concentrations were detected in the interior tissues (the carapace pulp and the bronchial muscle).

The statistical analysis showed significant inter-species differences observed with the ANOVA test (*p* < 0.001) for each element (Table 6).

**Figure 2:**
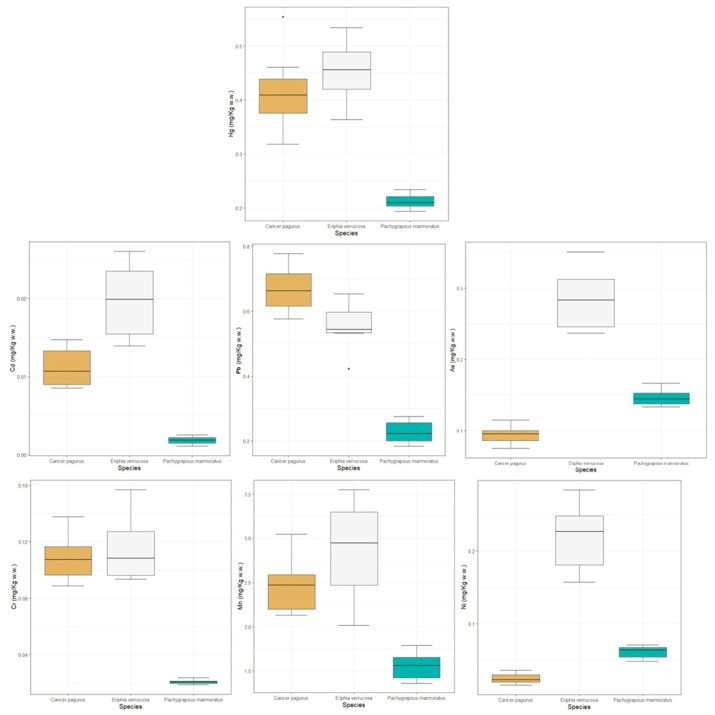
The box-and-whisker plot of trace metal and metalloid concentrations in the analyzed crab samples (μg g^−1^ w.w.) grouped by species. Diamonds indicate outliers.

In particular, Hg concentrations were significantly different between the three analyzed species (F = 44.57, *p* < 0.0001). Pb showed significant variation (F = 112.23, *p* < 0.0001), exhibiting markedly lower concentrations compared to the other species. Cd and As concentrations differed significantly among species (both *p* < 0.0001), with higher levels *in E. verrucosa* and *C. pagurus*. For Cr, differences were significant (F = 85.28, *p* < 0.0001), except between *C. pagurus* and *E. verrucosa* (*p* > 0.05) in the post hoc analysis. Mn showed significant differences mainly between *P. marmoratus* and the other two species, whereas the difference between *C. pagurus* and *E. verrucosa* was not significant. Finally, Ni concentrations varied significantly among all species (*p* < 0.001), with the highest mean values in *C. pagurus* (Figure 2).

**Figure 3:**
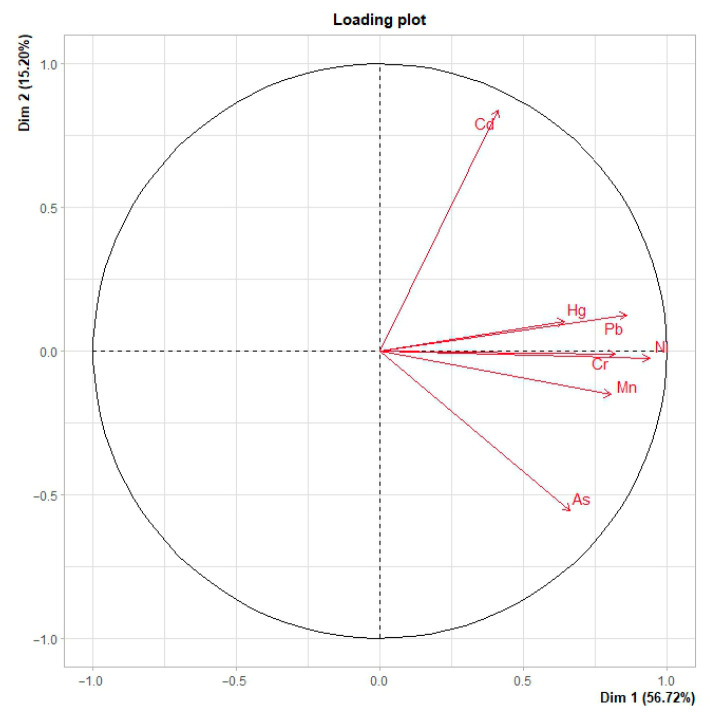
The loading plot ofPC1 vs. PC2 showing the contribution of trace metals and metalloids in the crab samples examined.

Principal component analysis (PCA) was performed by considering the quantitative concentrations of Hg, Pb, Cd, As, Cr, Mn and Ni measured in each individual sample as active variables (Figure 3). The first three principal components accounted for 83.6% of the total variance, with PC1, PC2 and PC3 explaining 56.7%, 15.3% and 11.7% of the variance, respectively. PC1 was strongly and inversely correlated with all metals, in particular Ni (r ≈ −0.94), Pb (r ≈ −0.86), Cr (r ≈ −0.83) and Mn (r ≈ −0.81), with As and Hg also contributing substantially (r ≈ −0.66 and r ≈ −0.65, respectively) and Cd to a lesser extent (r ≈ −0.41). Lower PC1 scores therefore correspond to higher overall metal burdens. PC2 captured a contrast between Cd and As, showing a strong positive correlation with Cd (r ≈ 0.84) and a marked negative correlation with As (r ≈ −0.56), while the remaining elements contributed only weakly to this axis. PC3 was dominated by Hg (r ≈ 0.72) with an opposite, moderate contribution of Mn (r ≈ −0.43), defining an additional dimension associated mainly with Hg-rich compositions.

The comparison by species showed that the centroids of *E. verrucosa* and *C. pagurus* were shifted towards lower PC1 values compared with *P. marmoratus*, indicating that, on average across tissues, the former two species exhibit a higher level of multi-element contamination. Among these species, *E. verrucosa* showed the highest overall metal accumulation, whereas *P. marmoratus* clearly had higher PC1 scores and thus the lowest integrated metal burden. Along PC2, *C. pagurus* displayed a distinctly positive centroid, while *E. verrucosa* and *P. marmoratus* were shifted towards negative values. This pattern indicates that, averaged across tissues, *C. pagurus* is relatively rich in Cd and comparatively low in As, whereas *E. verrucosa* and *P. marmoratus* exhibit the opposite tendency, with higher relative As for a given Cd level. The observations by anatomical compartment across species showed a marked separation along PC1. Carapace samples constituted the primary site of multi-element accumulation. Gill tissues displayed intermediate PC1 values, suggesting an appreciable but lower overall burden relative to the carapace. Conversely, the bronchial muscle and carapace pulp generally showed lower concentrations of all measured metals in soft tissues. Claws occupied an intermediate position near the origin of PC1, showing intermediate metal levels between the carapace and pulp. Along PC2, gills showed strongly positive scores, reflecting a composition relatively enriched in Cd and depleted in As, whereas the carapace showed negative PC2 values, with higher relative As. Claws also tended towards negative PC2 scores, further supporting an As-biased profile in hard appendicular structures.

**Figure 4:**
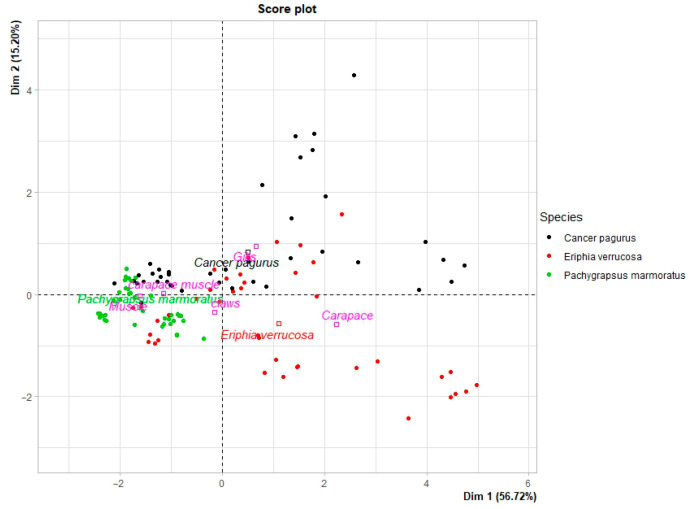
The PC1vs.PC2 score plot of trace metal and metalloid contents of the crab samples, according to the species.

Taken together, the PCA shows that PC1 describes a coherent increase in all measured metals, strongly differentiating the carapaces, particularly in *E. verrucosa* and *C. pagurus*, from the comparatively low-burden tissues of *P. marmoratus* and from soft tissues. The secondary axis (PC2) captures a Cd–As compositional gradient that discriminates *C. pagurus* and gill tissues (Cd-enriched) from the carapace and claws (As-enriched) (Figure 4).

**Figure 5:**
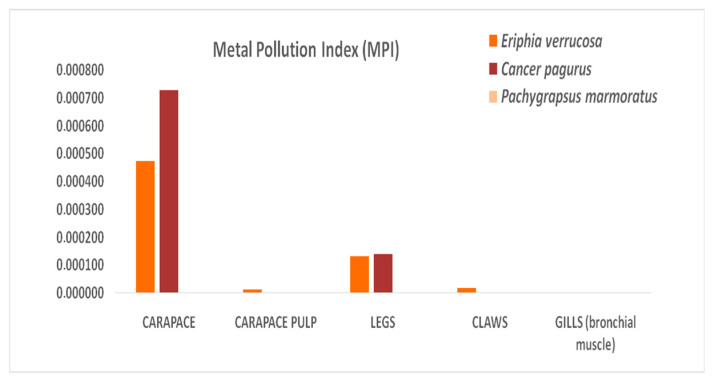
The Metal Pollution Index of the samples of *Eriphia verrucosa*, *Cancer Pagurus* and *Pachygrapsus marmoratus* from the Mediterranean coastlines of the Strait of Messina (Italy).

Considering all metals and metalloids found in various tissues, the Metal Pollution Index (MPI) showed values of <1 (safe level) in all three analyzed crab species (Figure 5).

**Figure 6:**
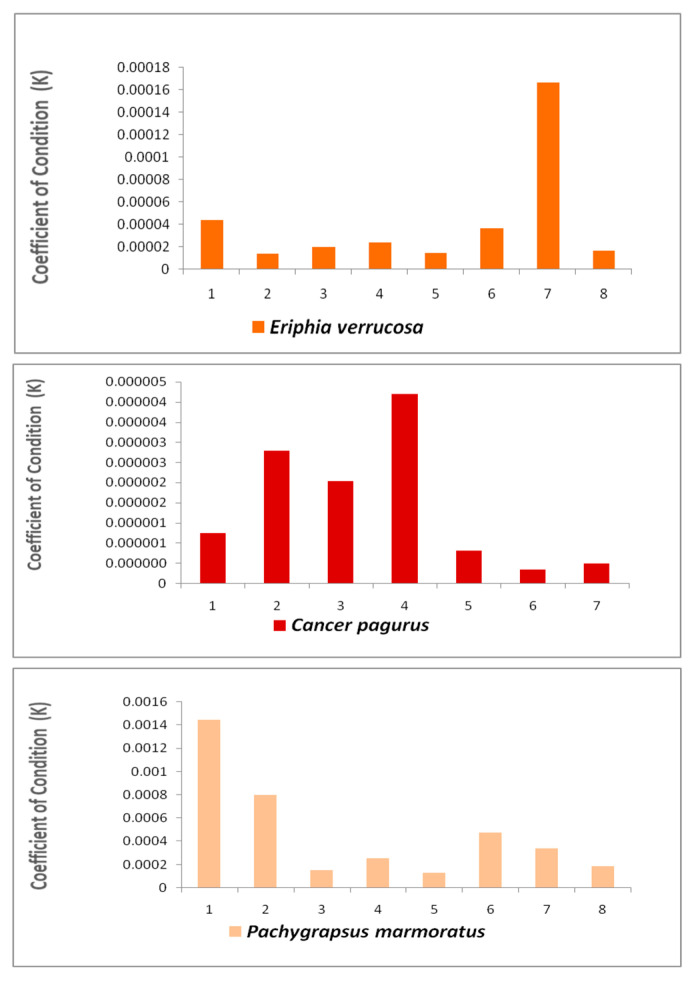
Coefficient of Correlation (K) in the analyzed crab samples (<1 safe level).

Regarding the parameters used to evaluate the health status of crabs in each sample of the three species analyzed the Coefficient of Condition (K) was <1, a value considered to be safe (Figure 6).

Finally, the EDI values were calculated from the metal and metalloid levels found in the carapace pulp (the edible part), and the results obtained are reported in Table 7. Very low EDI values were found for all the metals and metalloids, with higher values observed in *E. verrucosa* compared to *C. pagurus.*

## 4. Discussion

The exposure of crabs to heavy metals and metalloids is correlated to the presence of these contaminants in the marine environment where these aquatic species live and, particularly, to the mineral composition of the Mediterranean Sea basin [41]. In fact, in this natural semi-enclosed basin, pollutants are easily concentrated [1] and enter into marine species, with possible effects on their health status.

The metal and metalloid adsorption in crabs is linked to tissue affinity and species-specific biological differences. Considering the different crab tissues analyzed, the metal and metalloid content in the carapace, legs and claws could be due to a direct exposure in the marine habitat where crabs live and feed; moreover, the presence of residual levels in soft tissues (carapace pulp and bronchial muscle) is indicative of chronic exposure to these contaminants through ingestion from food, water, and sediment and/or their relative migration from the exoskeleton to the inner tissues and organs with possible toxicological risks [19]. In fact, from the obtained results, it is possible to observe that higher metal concentrations were present in the exterior parts when compared with the soft inner tissues (>carapace > gills claws > carapace pulp > bronchial muscle).

Among the three analyzed species, *E. verrucosa* and *C. pagurus* showed similar trends with higher metal levels than *P. marmoratus*. These differences could be due to the smaller size of *P. marmoratus* and also due to the higher ability of moving of the other two crab species.

Little data are present in the literature on the content of heavy metals and metalloids in the analyzed crab species. Regarding *E. verrucosa*, same studies documented the presence of heavy metals in warty crabs from the different areas of the Mediterranean Sea, such as the Campania region (Italy) [42] and Turkey [29,43]. In accordance with our results, these authors found the residual levels of Cd, Pb and Cr to be low and within the limits established for this seafood. Instead, several studies were conducted on the presence of metals in *C. pagurus* from different areas, such as Norway [33,44], France [45], and Nigeria [46], with particular attention given to Cd and Pb accumulation [33,47], probably because this species is commercially produced for human consumption. Finally, in relation to *P. marmoratus*, the study on metal residual levels was carried out in crab samples living in the Mediterranean Sea, particularly the Ligurian coast (Italy) [48], Lybia [49], and Azores archipelago [50]. Our results are similar to those obtained by Caliani et al. (2023) [48] in marbled crabs from the Ligurian coast of the Mediterranean Sea and Alvaro et al. (2016) [50], which found Mn and Zn as the most abundant metals. Considering the different parts analyzed, instead, other authors found higher heavy metal levels in the interior part of *P. marmoratus* than in the gills and other exoskeleton structures [51], contrary to our results.

For a complete toxicological evaluation of metals and metalloids exposure in crabs, the results obtained were used for both the ecological risk and the food safety assessment.

To assess metal and metalloid pollution in the Mediterranean areas where crabs lived, the MPI was used as a specific parameter of risk, reflecting the diffusion of metals from the aquatic environment into different tissues and organs of marine species [52]. The MPI obtained in this study showed a value of <1 (safe level) in all the analyzed samples, and this data is indicative of the low pollution level of the Mediterranean Sea, suggesting a insignificant health status risk for crab living in this marine ecosystem. The Coefficient of Condition (K), instead, a biometric parameter that expresses the habitat quality and marine species health status (according to organism’s development, specific energy level, physiological or pathological status of an animal, etc.), showed values of >1 in all analyzed crab species, indicating a good health status.

Considering that *E. verrucosa* and *C. pagurus* are highly appreciated sea-foods in Europe, their implications on consumer safety were evaluated through regulatory thresholds, such as MRLs, specific for each toxic metal and metalloid in the food matrix, and the calculation of the EDI. The presence of heavy metals and metalloids in soft tissues, such as the carapace pulp, which represents the edible part, is of particular interest because it may represent a potential risk for the consumer. For this reason, the European Food Safety Authority (EFSA) has set a maximum residual level for Hg and Pb in crabs (MRL of 0.50 mg/kg for both metals) [53] and, recently, also for As (MRL 0.10 mg/kg) [54]. From the results obtained, although the lowest concentrations of all metals were found in the carapace pulp (edible part), the residual Hg levels were above the MRL in *E. verrucosa* and *C. pagurus*, the Pb levels were higher than the limit in *C. pagurus*, and the As levels were above the MRL in all three analyzed crab species. Instead, the EDI values calculated for each metal in the carapace pulp were low and not significant for metal risk assessment and could be considered safe for human consumption.

The results obtained in this study could support the development of aquaculture, given the important role played by crabs, particularly *Eriphia verrucosa*, in the feeding of other farmed species (such as octopus, cuttlefish, sea bream, etc.). Currently, this practice is not carried out on a commercial scale, but it consists of the fishing and fattening in tanks to improve meat quality. In this practice, a system of sustainable management, considering the toxicological risks associated with pollutant exposure, could improve the quality of these seafoods for human consumption; at the same time, it supports the economy and prevents the decline in these species due to wild fishing and overfishing.

## 5. Conclusions

The presence of toxic metals and metalloids in the studied crab species is correlated to their exposure in the aquatic environment, although the observed MPI values suggested a low contamination level of the marine ecosystem in the Mediterranean Sea. An insignificant risk of metal exposure for the health status of these marine species is documented in the toxicological risk assessment, determined by the values of the Coefficient of Condition (K). Due to their specific biological features, crabs are, however, very sensitive to metal adsorption and their distribution from the exterior to the inner tissues and organs. Finally, considering the importance of these species as seafoods that are widely appreciated by consumers in the Mediterranean areas, the presence of Hg, Pb and As in the carapace pulp is above their MRLs, highlighting the potential toxicological risks and the importance of continuous monitoring studies to ensure seafood safety, albeit, the low values of EDI obtained could be indicative of safe human consumption.

## Figures and Tables

**Table 1:** The characteristics of three crab species found along the Sicilian coast of the Mediterranean Sea in the Strait of Messina (Italy).

Samples	N.	Sex	Carapace Length (cm)	Weight (g)
*Eriphia verrucosa* or “warty crab”	8	4 F 4 M	3.5–4	200–300
*Cancer pagurus * or “brown crab”	8	3 F 5 M	3.0–4.2	400–500
*Pachygrapsus marmoratus * or “marbled crab”	8	2 F 6 M	1.8–2.2	50–120

**Table 2:** The parameters of the analytical method used for metal determination.

Metals	Linearity (R^2^)	LOD (ηg g^−1^)	LOQ (ηg g^−1^)	Amount Added (ηg g^−1^)	Recovery (%)	RSD (%)
Hg	0.998	0.022	0.042	1–20	99.89	1.02
Pb	1.00	0.146	1.398	1–20	98.97	0.42
Cd	0.998	0.022	0.031	1–20	99.99	1.77
As	0.999	0.014	0.021	1–20	99.99	1.27
Cr	0.999	0.021	0.073	1–50	98.90	1.45
Mn	0.996	0.104	0.162	1–50	99.95	2.05
Ni	0.996	0.041	0.184	1–20	99.93	4.32

**Table 3:** The levels of toxic metals and metalloids in tissues/organs of *Eriphia verrucosa*, expressed as μg g^−1^ of individual measurements.

*Eriphia verrucosa*						
METAL		CARAPACE (μg g^−1^)	CARAPACE PULP (μg g^−1^)	GILLS (μg g^−1^)	CLAWS (μg g^−1^)	BRONCHIAL MUSCLE (μg g^−1^)
**Hg**	*M.V* ± *s.d.*	0.956 ± 0.166	0.451 ± 0.057	0.785 ± 0.176	0.764 ± 0.161	0.495 ± 0.108
**Pb**	*M.V* ± *s.d.*	1.678 ± 0.245	0.554 ± 0.046	1.063 ± 0.463	1.321 ± 0.422	0.291 ± 0.095
**Cd**	*M.V* ± *s.d.*	0.034 ± 0.006	0.019 ± 0.007	0.032 ± 0.007	0.004 ± 0.001	0.003 ± 0.001
**As**	*M.V* ± *s.d.*	0.672 ± 0.130	0.284 ± 0.038	0.410 ± 0.072	0.536 ± 0.097	0.284 ± 0.083
**Cr**	*M.V* ± *s.d.*	0.218 ± 0.083	0.114 ± 0.022	0.094 ± 0.027	0.071 ± 0.018	0.046 ± 0.011
**Mn**	*M.V* ± *s.d.*	6.146 ± 1.122	2.876 ± 0.568	3.148 ± 0.482	3.886 ± 0.977	1.724 ± 0.513
**Ni**	*M.V* ± *s.d.*	0.547 ± 0.125	0.219 ± 0.024	0.298 ± 0.081	0.245 ± 0.102	0.092 ± 0.024

**Table 4:** The levels of toxic metals and metalloids in tissues/organs of *Cancer pagurus*, expressed as μg g^−1^ of individual measurements.

*Cancer pagurus*						
METAL		CARAPACE (μg g^−1^)	CARAPACE PULP (μg g^−1^)	GILLS (μg g^−1^)	CLAWS (μg g^−1^)	BRONCHIAL MUSCLE (μg g^−1^)
**Hg**	*M.V* ± *s.d.*	0.809 ± 0.122	0.415 ± 0.075	0.804 ± 0.118	0.824 ± 0.179	0.705 ± 0.108
**Pb**	*M.V* ± *s.d.*	1.685 ± 0.477	0.671 ± 0.044	1.445 ± 0.336	1.478 ± 0.405	0.148 ± 0.068
**Cd**	*M.V* ± *s.d.*	0.025 ± 0.009	0.011 ± 0.003	0.052 ± 0.013	0.006 ± 0.002	0.007 ± 0.003
**As**	*M.V* ± *s.d.*	0.457 ± 0.085	0.094 ± 0.008	0.241 ± 0.051	0.181 ± 0.055	0.120 ± 0.082
**Cr**	*M.V* ± *s.d.*	0.231 ± 0.008	0.108 ± 0.012	0.104 ± 0.015	0.028 ± 0.009	0.043 ± 0.017
**Mn**	*M.V* ± *s.d.*	5.516 ± 0.805	2.471 ± 0.148	3.252 ± 0.624	2.885 ± 0.317	0.679 ± 0.264
**Ni**	*M.V* ± *s.d.*	0.491 ± 0.127	0.025 ± 0.008	0.338 ± 0.062	0.251 ± 0.089	0.069 ± 0.018

**Table 5:** The levels of toxic metals and metalloids in tissues/organs of *Pachygrapsus marmoratus*, expressed as μg g^−1^ of individual measurements.

*Pachygrapsus marmoratus*						
METAL		CARAPACE (μg g^−1^)	CARAPACE PULP (μg g^−1^)	GILLS (μg g^−1^)	CLAWS (μg g^−1^)	BRONCHIAL MUSCLE (μg g^−1^)
**Hg**	*M.V* ± *s.d.*	0.512 ± 0.085	0.212 ± 0.013	0.477 ± 0.172	0.558 ± 0.051	0.234 ± 0.056
**Pb**	*M.V* ± *s.d.*	0.429 ± 0.126	0.226 ± 0.018	0.458 ± 0.077	0.290 ± 0.073	0.115 ± 0.035
**Cd**	*M.V* ± *s.d.*	0.005 ± 0.001	0.002 ± 0.0007	0.004 ± 0.001	0.004 ± 0.001	0.012 ± 0.003
**As**	*M.V* ± *s.d.*	0.279 ± 0.071	0.146 ± 0.012	0.219 ± 0.044	0.086 ± 0.007	0.012 ± 0.002
**Cr**	*M.V* ± *s.d.*	0.046 ± 0.013	0.021 ± 0.008	0.064 ± 0.053	0.019 ± 0.005	0.032 ± 0.006
**Mn**	*M.V* ± *s.d.*	2.169 ± 0.234	1.549 ± 0.150	2.859 ± 0.468	1.904 ± 0.407	1.625 ± 0.292
**Ni**	*M.V* ± *s.d.*	0.154 ± 0.053	0.061 ± 0.007	0.133 ± 0.025	0.076 ± 0.014	0.045 ± 0.003

**Table 6:** Inter-species differences in crab samples.

Element	N for Each Species	*p*-Value
Hg	8	2.77 × 10^8^
Pb	8	6.15 × 10^3^
Cd	8	1.04 × 10^7^
As	8	3.03 × 10^4^
Cr	8	8.30 × 10^4^
Mn	8	3.07 × 10^10^
Ni	8	4.11 × 10^3^

**Table 7:** The residual levels of metals and metalloids expressed as EDI (µg/g/day).

CarapacePulp of *Eriphia verrucosa*		CarapacePulp of *Cancer pagurus*	
Metal	EDI	Metal	EDI
Hg	0.000097	Hg	0.000045
Pb	0.000119	Pb	0.000048
Cd	0.000004	Cd	0.000000
As	0.000061	As	0.000031
Mn	0.000616	Mn	0.000332
Cr	0.000024	Cr	0.000004
Ni	0.000047	Ni	0.000013

## Data Availability

Data and results related to this study are original and included in the article.
